# Employments for Women

**Published:** 1891-07-25

**Authors:** 


### Employments for Women.

### II.—TYPE-WRITING FOR LADIES.

"What shall we do with our daughters?" is a question that has been asked, and will continue to be asked, by many a one besides Mr. Corney Grain; and to many an anxious parent the answer has opened a door of hope—"Why, make type-writers of them!" And, indeed, there seems to be no reason against type-writing's proving a very suitable employment for women of education and gentle breeding. While the mere type-writing is and must be mechanical, there is plenty of scope, and plenty of need, for intelligence; and it is obvious that general information, not to speak of a knowledge of various languages, must be of great use to the type-writer. There is something, then, for the brain to work upon, as well as for the hand; and when once a certain amount of manual dexterity has been obtained, the worker must be at liberty to glean a considerable amount of interest and amusement for herself out of certain of the tasks set before her.

Let us take a look at a lady type-writers' office, where three sisters are joint heads of the concern, working themselves, and employing several ladies under them. In the largest of the three rooms, several machines are busily at work. One girl is only a beginner, and is anxiously trying to learn the position of the keys on which are marked the various letters of the alphabet, arranged, not like our own A B C, but according to a system which wisely places the letters most often needed in the most get-at-able places—that is to say, in the middle. Another girl is perhaps sighing a little over a builder's specification, which she cannot make interesting to herself, try as she will; but her neighbour has before her a manuscript chapter of a thrilling novel, now coming out in monthly parts; and thereout, as her fingers rapidly press the keys, sucks she no small advantage. To another is entrusted the private journal kept by two ladies making a tour in the East, which she is carefully putting into type, leaving spaces here and there for the photographs which are to embellish the book. These will be put in, and the book tastefully bound, before it is returned to its authors, so that they will have no further trouble about it.

But at this minute the door which leads into the "Chief's" private room opens; and she—the Chief—looks in. "I want someone to go into the further room, quick!" she says. "Mr. B. has just looked in, and wants us to take down an article for him against time; it is going into the *Evening Mail* to-night, I believe." And as one of the girls hurries off into the further room, there to work away at the machine which stands waiting, while the busy journalist strides up and down the room, pouring forth a torrent of words—the Chief shuts the door behind her, and says, with a twinkle in her eye—"And, girls, who do you think is here? Who but the old gentleman whose manuscript has baffled us all? My last letter has actually lured him from his den, and now he declares that if we're all such dunderheads, he must tackle the manuscript himself, and read it out to us. Who'll volunteer for the task?" There is a general laugh at this, for that manuscript has been the whole round of the room, and has finally been cast aside in despair. When it first made its appearance, the Chief was obliged to say that it could not be undertaken at the usual terms, on account of its illegibility; finally, she had to state that it could not be undertaken at any price, for not one of the staff could read a word of it. "Oh, let me go!" says one of the girls, springing up. "It'll be the greatest fun to see him confronted with it. If he reads it—why, he'll be cleverer than I take him for!"

But to come back to the more practical side of the matter. "Is type-writing an employment which one can live by? and is it expensive to learn?" are questions which are sure to be eagerly asked by some. To answer the latter question first: type-writing is taught—in certain offices, at any rate—for the fee of two guineas, the pupil having the use of a machine, &c., for six weeks daily, or for half a day for twelve weeks. It is considered that at the end of this time she should know all that can be taught, though of course speed and finish will only come by longer experience. She should then begin to earn for herself, the usual practice being, we believe, to give her half what she earns—that is to say, if one-and-threepence per thousand words is charged in the office, half should go to the owner of the machine, and half to the worker.

As to how much can be earned by type-writing, this of course varies greatly according to the efficiency of the "operator." Some are slow with their fingers; some lack general knowledge; others prove deficient, somehow, in what we may call *sensibleness*, and lose much time in asking for directions, or correcting stupid mistakes; so that of two operators who have been a year at the work, it may well happen that one is only taking nine shillings a week, while the other earns thirty. As a rule, we think, a type-writer should be able to earn from thirty shillings to two pounds a week, working from ten to five or six. This cannot be called high remuneration; but type-writers should look to themselves most carefully, and jealously set their faces against temptations to undersell each other, or the rate of payment will fall lower still. There is really no reason—except the force of competition, and the tendency of the world to consider women's work as of low money value—why type-writing should not command a good price, for, as has been seen, it is by no means unskilled labour. If it be true, as we fear it is, that some type-writing offices make it a rule only to take such *employées* as have other means of subsistence, and can therefore work for less pay, we can only say that they deserve to be pilloried, and that anyone who would show up such conduct would deserve the thanks, not only of type-writers, but of the public in general.

We may mention that shorthand is a most useful adjunct to type-writing; and that girls who can take down notes in shorthand, and afterwards type-write letters from the notes, make excellent secretaries.

